# UCHL5 suppresses thyroid carcinoma progression via ZRANB1 stabilization and ferroptosis regulation

**DOI:** 10.1080/15384047.2026.2663610

**Published:** 2026-04-27

**Authors:** Wenxia Xiao, Yu Ding, Xuesuo Wu, Fenglian Que, Baozhi Wang, Shanshan Hong, Fenghua Zhang

**Affiliations:** a Endocrinology Department, the Second Affiliated Hospital of Guangzhou Medical University, Guangzhou, China; b General Surgery Department, the Second Affiliated Hospital of Guangzhou Medical University, Guangzhou, China; c Department of Respiratory Medicine, the Second Affiliated Hospital of Guangzhou Medical University, Guangzhou, China

**Keywords:** UCHL5, thyroid cancer, ZRANB1, ferroptosis, deubiquitination

## Abstract

**Objective:**

This study investigated the role of UCHL5 in thyroid carcinoma (THCA) progression, focusing on its tumor-suppressive mechanisms and regulation of ferroptosis.

**Methods:**

We performed multi-omics analysis of TCGA and GEO datasets and validated the findings using clinical samples. The CRISPR/Cas9-mediated knockout and stable overexpression in THCA cell lines were constructed, followed by comprehensive functional assays including co-immunoprecipitation (Co-IP), ubiquitination analysis, xenograft tumor models, and ferroptosis sensitivity tests using erastin/ferrostatin-1 with BODIPY C11 lipid ROS measurement. Weighted gene co-expression network analysis (WGCNA) was performed to identify hub genes and to analyze associated pathways.

**Results:**

Clinical data revealed significant downregulation of UCHL5 in advanced thyroid cancers, particularly in lymph node metastases. UCHL5 knockout markedly accelerated cell proliferation and xenograft tumor growth, while its overexpression suppressed both. Mechanistically, we identified a direct interaction between UCHL5 and ZRANB1 through co-IP, with UCHL5 extending the protein half-life of ZRANB1 by over 2-fold. In ferroptosis regulation, erastin treatment (10 μM) revealed that UCHL5 overexpression enhanced sensitivity, while UCHL5 knockout conferred significant protection, accompanied by altered GSH levels, Fe²⁺ accumulation, and lipid ROS production. Western blot analysis revealed that UCHL5 upregulated ZRANB1 and downregulated SLC7A11/GPX4 expression.

**Conclusion:**

This study demonstrates that UCHL5 acts as a critical tumor suppressor in thyroid cancer by stabilizing ZRANB1 through deubiquitination and regulating ferroptosis via the SLC7A11-GPX4 axis.

## Introduction

Thyroid carcinoma (THCA) represents the most prevalent endocrine malignancy worldwide, with 586,000 new cases reported in 2020 and an age-standardized incidence rate of 7.7 per 100,000 individuals.^1^ Among its histological subtypes, papillary thyroid carcinoma (PTC) accounts for approximately 80% of diagnoses.^2^ Although 10-y survival exceeds 95% for patients with localized PTC, prognosis worsens significantly upon distant metastasis.^3^ BRAFV600E mutations are common in recurrent and treatment-refractory THCA, detected in up to 30% of relapsed cases.^4^^,^^5^ Additionally, the Cancer Genome Atlas (TCGA) and other large genomic investigations show that TERT promoter mutations are independent adverse prognostic markers associated with a 7.1-fold increase in THCA-specific mortality.^6^ Thus, defining the prognostic utility and therapeutic potential of molecular targets in advanced THCA care is essential for driving the development of precision interventions.

The proteasome-related UCHL5 deubiquitinase has important functions in cellular proteostasis, as evidenced by its capacity to edit ubiquitin, inhibit endopeptidase and regulate proteasome activity.^7^ UCHL5 is involved in diverse biological processes, ranging from metabolic regulation to developmental signaling pathways,^7-10^ and growing evidence supports its oncogenic role in various cancers.^7^^,^^11^^,^^12^ For instance, UCHL5 drives hepatocellular carcinoma progression through Wnt/β-catenin-mediated metabolic reprogramming,^8^ sustains pancreatic cancer stemness by stabilizing ELK3,^9^ promotes bladder cancer chemoresistance by suppressing ferroptosis. Despite these insights in other cancer types, the role and underlying mechanisms of UCHL5 in THCA remain largely unexplored.

The objective of this study is to explore the biological and clinical significance of UCHL5 in THCA employing integrated multi-omics analyses, functional experiments, and a mechanistic study. Special attention is given to its potential involvement in mediating resistance to ferroptosis through the zinc finger RANBP2-type containing 1 (ZRANB1)-solute carrier family 7 member 11 (SLC7A11) axis. Elucidating UCHL5-mediated tumor suppression may provide insights into THCA progression and reveal novel therapeutic strategies.

## Methods

### Data acquisition and preprocessing

Transcriptome profiles and clinical characteristics of patients (age, sex, TNM classification, and overall survival) were obtained from the Cancer Genome Atlas (TCGA-THCA dataset using GDC portal (https://portal.gdc.cancer.gov/)). To enhance the robustness of the analysis, three public microarray datasets, GSE3467 (9 matched pairs), GSE60542 (27 matched pairs), and GSE33630 (44 matched pairs) were downloaded from the GEO database.^13^

### Differential expression analysis

We obtained transcriptomic and clinical data, including age, sex, TNM stage, and survival – from the TCGA-THCA dataset via the GDC portal (https://portal.gdc.cancer.gov/). To strengthen the robustness of the analysis, three publicly available microarray datasets, including GSE3467 (9 matched pairs), GSE60542 (27 matched pairs), and GSE33630 (44 matched pairs), were obtained from the Gene Expression Omnibus (GEO) datasets.^13^ Each dataset comprises paired thyroid tumor and adjacent normal tissues, providing independent external cohorts for validation.

### Immune infiltration and immune checkpoint analysis

The GSVA R package^14^ was used to determine immune cell infiltration levels through single-sample gene set enrichment analysis (ssGSEA). This study assessed twenty-four populations of immune cells, comprising natural killer (NK) cells, cytotoxic T lymphocytes (CTLs), and dendritic cells. We employed Pearson’s correlation or Spearman’s correlation analyses, according to the data distribution, to evaluate the relationship between UCHL5 expression and immune cell infiltration. Graphs were generated using ggplot2 and ggpubr packages.^15^

Additionally, the relationship between *UCHL5* expression and a panel of immune checkpoint molecules – including PD-1, CTLA-4, CD160, and ARHGEF5 – was explored to assess its potential role in immune escape. The correlation results were visualized as a heatmap to highlight expression trends across the samples.

### Single-cell RNA sequencing analysis

Using unsupervised clustering and marker-based annotation, distinct cell populations, including malignant epithelial cells, fibroblasts, CD8⁺ T lymphocytes, dendritic cells, and monocytes/macrophages, were identified.^16^ We utilized Uniform Manifold Approximation and Projection (UMAP)^17^ to achieve dimensionality reduction and visualization of cellular distributions. The expression profiles of *UCHL5* in the identified cell types were calculated using the Seurat R package. Cell-type-specific correlation analyses were performed to study the immunoregulatory potential of UCHL5.

### Association with clinicopathological features

Based on the median UCHL5 transcript level, patients from the TCGA-THCA cohort were divided into high- and low-expression groups. The Wilcoxon rank-sum test or the Kruskal-Wallis test was used to determine associations between UCHL5 expression, age, sex, TNM grade, and overall clinical stage.

### Tissue sample collection and ethical considerations

The specimens of formalin-fixed paraffin-embedded tissue of the thyroid were taken post-surgery between January 2024 and December 2024. All cases were confirmed by HPE (histopathological examination), and only those that had more than 50% tumoral cellularity were included in the study following review by two pathologists.

All procedures involving human participants were conducted by the ethical standards of the institutional and national research committees, as well as the 1964 Helsinki Declaration and its subsequent amendments. Written informed consent was obtained from each patient prior to the use of their tissue samples for research purposes. Approval for the use of patient samples and clinical data was obtained from the Institutional Review Board of The Second Affiliated Hospital of Guangzhou Medical University (2024-hg-ks-37).

### Immunohistochemistry (IHC) analysis

IHC was performed using the streptavidin–peroxidase method. Briefly, tissue sections were deparaffinized, rehydrated, and subjected to heat-induced antigen retrieval in citrate buffer (pH 6.0). Endogenous peroxidase activity was quenched with 3% hydrogen peroxide. Following antigen retrieval, the sections were incubated overnight at 4 °C with a primary antibody specific for UCHL5 (cat. no. ab192616, 1:300, Abcam, USA). Detection was achieved using a horseradish peroxidase (HRP)-conjugated secondary antibody (cat. no. ab6721, 1:500, Abcam, USA), followed by chromogenic development with 100 μL 3,3 μL followed by ch (DAB, cat. no. 7411-49-6, maokangbio, China). Nuclei were counterstained with hematoxylin. Protein expression levels were semiquantitatively assessed by evaluating both the proportion and staining intensity of positively labeled tumor cells. An immunoreactivity score (H-score) was calculated using the formula:

IHCscore∑cpercentageofcellsateachstainingintensity×intensityvalue).where staining intensity was classified as: Score 0: No staining or <10% of tumor cells with weak membranous staining; Score 1+: Faint or barely perceptible membrane staining in 10%–25% of tumor cells; Score 2+: Weak to moderate complete membrane staining observed in 26%–50% of tumor cells; Score 3+: Strong complete membrane staining in >50% of tumor cells.

### Cell culture

Human THCA cell lines: FTC-133 (cat. no.1101HUM-PUMC000687), BHT-101 (cat.no.3101HUMSCSP544), B-CPAP (cat.no.3101HUMSCSP543), and ATC-1(3101HUMSCSP541) were obtained from the Cell Resource Centre of Wuhan University. 8505 C (cat. No. BFN60808779), and TPC-1 (cat.no.BFN680325) were obtained from the Shanghai Cell Bank, China. All cell lines were cultured in high-glucose Dulbecco's Modified Eagle Medium (Gibco, Cat. No. 11965092), supplemented with 10% fetal bovine serum (Gibco, Cat. No. 10099141), 100 U/mL penicillin/streptomycin (Gibco, Cat. No. 15140122), and 1.5 mg/L L-glutamine (Gibco, cat. No. 25030081). Cells were maintained at 37 °C in a humidified atmosphere containing 5% CO₂. All cell lines were authenticated by short tandem repeat (STR) profiling and tested negative for mycoplasma contamination using a PCR-based detection kit (cat. No. G2380, Servicebio, China) prior to experiments.

### CRISPR/Cas9 knockout UCHL5 in THCA cells

To generate UCHL5-knockout THCA cell lines, we designed sgRNAs targeting the human UCHL5 genomic sequence (Ensembl). Potential sgRNA targets proximal to exon–intron boundaries were predicted using the CRISPOR platform (http://crispor.tefor.net/), and further filtered for on-target efficiency and minimal off-target potential using CCTop (https://cctop.cos.uni-heidelberg.de). Two sgRNAs exhibiting optimal specificity profiles were synthesized (Suzhou Synthesis Biosciences, China) and cloned into the lentiviral backbone LV-U6>sgRNA-CMV>hCas9/P2A/EGFP. Recombinant plasmids were validated via colony PCR and confirmed through Sanger sequencing.

Lentiviral particles were generated by transient transfection of HEK293T packaging cells with the sgRNA constructs and helper plasmids using 10 μL Lipofectamine 3000 (cat. no. L3000015, Thermo Fisher Scientific). Supernatants containing viral particles were collected at 48 and 72 h post-transfection, filtered, and used to transduce FTC-133 and TPC-1 THCA cell lines. Transduction efficiency was assessed by detecting EGFP fluorescence. Fluorescent-positive cells were subsequently sorted using fluorescence-activated cell sorting (FACS) and individually seeded into 96-well plates to derive monoclonal populations.

Single-cell clones were progressively expanded and screened for *UCHL5* knockout via RT-PCR and Sanger sequencing of the target loci. Two successfully edited monoclonal lines, originating from FTC-133 and TPC-1, were confirmed to lack functional *UCHL5* expression and were subsequently employed in functional and mechanistic studies.

### Construction of UCHL5 overexpressing THCA cells

The full-length coding sequence of human UCHL5 was amplified via PCR and subsequently cloned into the p3×FLAG-CMV expression vector, which encodes an *N*-terminal FLAG epitope tag. The fidelity and orientation of the insert were verified by Sanger sequencing. The resulting plasmid construct (FLAG-UCHL5) was introduced into FTC-133 and TPC-1 THCA cells via transient transfection using Lipofectamine 3000 (Thermo Fisher Scientific) according to the manufacturer's protocol.

Following 48 h of transfection, G418 (cat. no. G5013, Geneticin; Gibco) was applied at a final concentration of 500 μg/mL to select for stably transfected clones over 10–14 d. Surviving colonies were individually picked, expanded, and screened for *UCHL5* overexpression. Protein expression was confirmed by Western blotting using both anti-FLAG and anti-UCHL5 antibodies.

### Cell proliferation assay

Cell proliferation was evaluated using the Cell Counting Kit-8 (cat. no. CK04-10, CCK-8; Dojindo Laboratories, Japan), following the manufacturer's instructions. THCA cells were suspended in complete growth medium at a concentration of 1 × 10^4^ cells/mL and plated into 96-well culture plates at 100 μL per well. Cell viability was monitored at 0, 24, 48, and 72 h post-seeding. At each time point, 10 μL of the CCK-8 reagent was added to each well and incubated at 37 °C for 4 h. The absorbance at 450 nm, which reflects cellular metabolic activity, was measured using a microplate spectrophotometer (Bio-Rad, USA).

### Colony formation assay

In order to assess the clonogenic potential of the THCA cells, a single-cell suspension of the cells was prepared using trypsinization and plated in 6-well tissue culture plates at a density of 500 cells/well using DMEM. Cells were kept under standard culture conditions (37 °C, 5% CO₂) for 10–14 d. Following the visible formation of colonies, the wells were rinsed gently using phosphate-buffered saline (PBS). Wells were then fixed with 4% paraformaldehyde for 20 min. Then, the wells were stained with 0.1% crystal violet solution (Bio-Rad, USA; cat. no. 1705061) for 30 min at room temperature. A light microscope was used to manually count colonies with over 50 cells.

### Co-immunoprecipitation (Co-IP)

Plasmids encoding Flag-tagged UCHL5 and HA-tagged ZRANB1 were transiently transfected into HEK293T cells through Lipofectamine 3000 (Thermo Fisher Scientific, USA; Cat. No. L3000015) as per the manufacturer’s instructions. The cells were harvested after 48 h and lysed on ice using immunoprecipitation (IP) lysis buffer from Thermo Fisher Scientific (cat. No. 87787, USA). The lysates were left on ice for 30 min and then centrifuged for 15 min at 13,000  rpm and at 4 °C to obtain clarified supernatants.

For immunoprecipitation, pre-equilibrated agarose-conjugated 20  μL anti-Flag (cat. No.: HY-K0236, MMonmouth Junction, NJ, USA), or 20 μL anti-HA antibodies (cat. no. 20786ES03, Yeasen, Shanghai, China) were added to the supernatants and incubated at 4 °C with gentle rotation for 2 h or overnight. The beads were then washed 4–5 times with cold lysis buffer to eliminate nonspecific binding. Bound proteins were eluted by boiling the beads in 2× SDS sample buffer for 5 min at 95 °C. Eluted protein complexes were subjected to SDS–PAGE, followed by immunoblotting using anti-Flag and anti-HA antibodies to verify the physical interaction between UCHL5 and ZRANB1.

### Xenograft tumor model

The protocol was approved by the Institutional Animal Care and Use Committee (IACUC) of Guangzhou Miles Biosciences, China (Approval No. IACUC-MIS2023061), an AAALAC-accredited facility. The animal facility is independent of the authors' institution, and the experiments were carried out by trained personnel at this commissioned facility under their animal license (SYXK(Yue)2022-0301).

A total of eighteen female BALB/c nude mice (6–8 weeks old, specific pathogen-free) were obtained from the Guangdong Weitong Lihua Laboratory Animal Technology Co., Ltd, China. Mice were housed in a controlled environment (12-h light/dark cycle, 22 ± 2 °C, 50 ± 10% humidity) with free access to autoclaved food and water. After one week of acclimatization, the mice were randomly assigned to three groups (WT, KO1, KO2; *n* = 6 per group).

For the tumor inoculation procedure, mice were briefly anesthetized via inhalation of 2% isoflurane (RWD Life Science, China) in oxygen delivered through a precision vaporizer. TPC-1 cells in the logarithmic growth phase were harvested by trypsinization and resuspended at a final concentration of 2 × 10⁷ cells/mL. Each mouse received a subcutaneous injection of 0.2 mL cell suspension (equivalent to 4 × 10⁶ cells) into the right axillary region. Tumor dimensions and body weight were measured every other day using calipers and an electronic scale, respectively. Tumor volume was calculated according to the standard formula: volume = ½ × length × width². All mice were sacrificed by cervical dislocation. Tumors were then excised, photographed, and weighed.

### Detection of lipid peroxidation by flow cytometry

FTC-133 wild-type (WT), UCHL5 knockout clones (KO1 and KO2) were seeded into 6-well plates and treated for 24 h with either erastin (cat. no. 54037ES05, 10 μM, Yeason, Shanghai, China), erastin combined with ferrostatin-1 (cat. no. 54020ES, 1 μM, Yeason, Shanghai, China), or an equivalent volume of DMSO as a control. Following treatment, cells were harvested by trypsinization and incubated with 2 μM BODIPY 581/591 C11 dye (Thermo Fisher Scientific, Cat# D3861) at 37 °C for 30 min in the dark to label lipid peroxidation products. After staining, cells were immediately analyzed using flow cytometry (BD FACSCanto II). Changes in fluorescence intensity were quantified to assess lipid reactive oxygen species (ROS) accumulation. Data acquisition and analysis were performed using FlowJo software (BD Biosciences).

### Intracellular glutathione (GSH) detection

Intracellular total GSH content was measured using a GSH Assay Kit (Beyotime, China; cat. No. S0052) according to the manufacturer’s instructions. Briefly, UCHL5-overexpressing B-CPAP cells and UCHL5-knockout FTC-133 cells (including wild-type and Mock controls) were seeded in 6-well plates and cultured to 80% confluence. After repeated freezing and thawing, the lysates were centrifuged, and the supernatants were collected. The reaction system was prepared by mixing 50 μL of supernatant with 50 μL of reaction buffer, and the absorbance at 412 nm was measured using a microplate spectrophotometer (Bio-Rad, USA) after incubation at 37 °C for 15 min. GSH concentration was calculated according to the standard curve and normalized to the total protein concentration of each sample.

### Intracellular ferrous Ion (Fe²⁺) detection

Intracellular Fe²⁺ levels were detected using the FerroOrange fluorescent probe (Cell Signaling; cat. No. 36104). UCHL5-overexpressing B-CPAP cells and Mock control cells were seeded in confocal dishes and cultured to 70% confluence. The culture medium was discarded, and cells were incubated with 5 μM FerroOrange probe in serum-free DMEM at 37 °C for 20 min in the dark. The fluorescent images were captured using an automated cell imaging analysis system (Olympus, Japan). The fluorescence intensity of Fe²⁺ was quantified using ImageJ software.

### Western blot

Cells were lysed in 200 μL RIPA buffer (Beyotime, Cat. No. P0013B). The supernatant was collected and subjected to protein quantification. Next, mix equal parts of overall protein was mixed with the SDS sample buffer and then boiled denatured at 95 °C. After that, the samples were added to the 12% SDS-PAGE gels. The protein transfer was further made on polyvinylidene difluoride (PVDF) membranes (Millipore, USA). That was later blocked with the non-fat milk having 5% concentration. Membranes were incubated overnight at 4 °C with primary antibodies against UCHL5 (Abcam, ab192616, 1:1000), Flag (Sigma–Aldrich, F1804, 1:2000), ZRANB1 (ProteinTech, 12071-1-AP, 1:1000), SLC7A11 (Cell Signaling Technology, 12691S, 1:1000), GPX4 (Abcam, ab125066, 1:1000), and GAPDH (ProteinTech, 60004-1-Ig, 1:5000). After three washes in TBST, membranes were incubated with horseradish peroxidase (HRP)-conjugated secondary antibodies (Lianke Bio, China; dilution 1:3000) for 2 h at room temperature. Protein bands were visualized using 200 μL enhanced chemiluminescence (ECL) reagents (Bio-Rad, cat. No. 1705061) and captured with a chemiluminescence imaging system.

### Statistical analysis

All statistical analyses were performed using GraphPad Prism version 10.0 (GraphPad Software, USA) and R software (version 4.0). Quantitative data are presented as mean ±  standard deviation (SD). For comparisons between two groups, either unpaired or paired two-tailed Student’s *t*-tests were applied, depending on the experimental design. Differences among multiple groups were assessed using one-way analysis of variance (ANOVA), followed by Tukey’s post-hoc test for pairwise comparisons.

Pearson's correlation coefficients were calculated to evaluate linear associations between continuous variables. A *P*-value < 0.05 was considered statistically significant. Statistical significance in graphical presentations is denoted as follows: *P* < 0.05. All experiments were performed at least in triplicate.

## Results

### Downregulation of UCHL5 in THCA tissues

**Figure 1.**
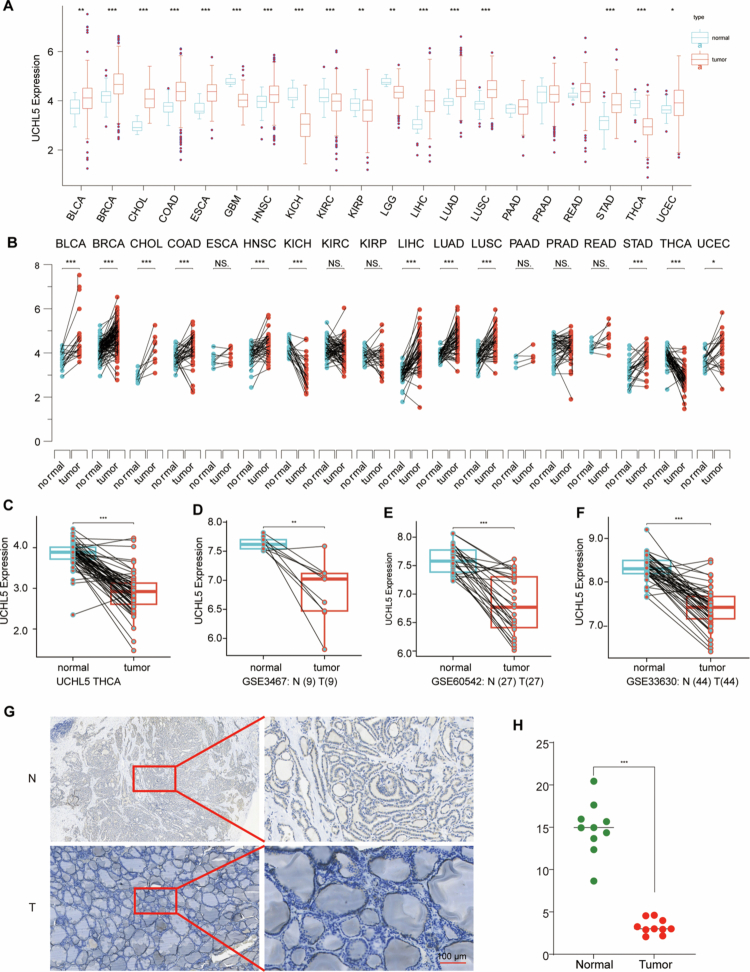
UCHL5 is significantly downregulated in THCA tissues at both transcript and protein levels. (A) Boxplot of UCHL5 mRNA expression in tumor versus normal tissues across multiple cancer types based on the TCGA pan-cancer dataset. (B) Paired expression analysis of UCHL5 in matched tumor and adjacent standard samples from TCGA cohorts, highlighting differential expression across individual cancer types. (C) UCHL5 expression in THCA versus normal thyroid tissues in the TCGA-THCA dataset. (D) UCHL5 expression in thyroid cancer (T) and normal (*N*) tissues from the GSE3467 dataset (*N* = 9, T = 9). (E) UCHL5 expression in thyroid cancer (T) and normal (*N*) tissues from the GSE60542 dataset (*N* = 27, T = 27). (F) UCHL5 expression in thyroid cancer (T) and normal (*N*) tissues from the GSE33630 dataset (*N* = 44, T = 44). (G) Representative immunohistochemical images showing UCHL5 protein expression in paired normal (*N*) and tumor (T) thyroid tissues (scale bar = 100 μm). (H) Quantitative analysis of IHC staining intensity for UCHL5 in tumor versus normal thyroid tissues.

Initial analysis of TCGA pan-cancer data identified tissue-specific dysregulation of UCHL5 across human malignancies, with THCA showing the most pronounced downregulation compared to normal tissues (Figure 1A,B). In the TCGA-THCA cohort (*n* = 568), we confirmed significant downregulation of UCHL5 at the transcript level (*p* < 0.001, Figure 1C). This observation was further validated via analysis of three independent Gene Expression Omnibus (GEO) datasets: GSE346 (9 tumor-normal pairs, *p* = 0.008), GSE60542 (27 pairs, *p* = 0.003), and GSE33630 (44 pairs, *p* < 0.001) (Figure 1D–F).

At the protein level, IHC analysis of matched tumor-normal pairs revealed a substantial reduction of UCHL5 expression in malignant thyroid tissues (*p* < 0.001) (Figure 1G–H). The downregulation observed across all datasets strongly supports UCHL5 as a potential tumor suppressor in THCA.

### UCHL5 downregulation associates with advanced disease features in THCA

**Figure 2.**
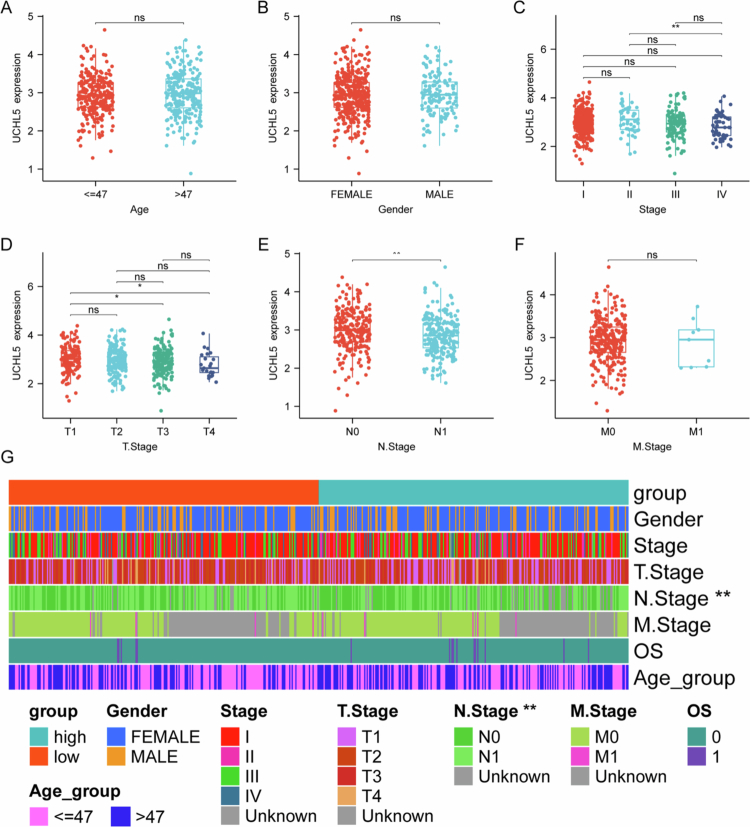
Association of UCHL5 expression with clinicopathological features in THCA. (A) UCHL5 mRNA expression in patients aged 5 expression with >47 y. (B) UCHL5 expression stratified by gender (female vs. male). (C) UCHL5 expression across clinical stages (I–IV). (D) UCHL5 expression across tumor stages (T1–T4). (E) Comparison of UCHL5 levels between patients with (N1) and without (N0) lymph node metastasis. (F) Comparison of UCHL5 levels between patients with (M1) and without (M0) distant metastasis. (G) Distribution of clinicopathological characteristics (gender, age group, clinical stage, T stage, *N* stage, M stage, and overall survival status) in UCHL5-high versus UCHL5-low expression groups, defined by the median expression level.

UCHL5 expression did not correlate with age (≤47 vs. >47 y) or sex (male vs. female) (Figure 2A,B). However, it decreased progressively with advancing disease stage (Figure 2C). Particularly, the UCHL5 expression was significantly lower in tumors at stage III than earlier at stage I (Figure 2C). Moreover, the T3 tumors exhibited a significant reduction when compared with T1/T2 stages (Figure 2D). In particular, UCHL5 expression was significantly reduced in cases with lymph node metastasis (N1) compared to node-negative (N0) ones (Figure 2E). However, a difference in distant metastasis was not seen (Figure 2F). Analysis of stratification suggested that more patients with lymph node metastasis were enrolled in the low- expression UCHL5 group (Figure 2G). Therefore, these findings suggest that low UCHL5 expression is associated with aggressive clinicopathological features in THCA.

### UCHL5 expression correlates with immune cell infiltration and immune checkpoint marker expression in THCA

**Figure 3.**
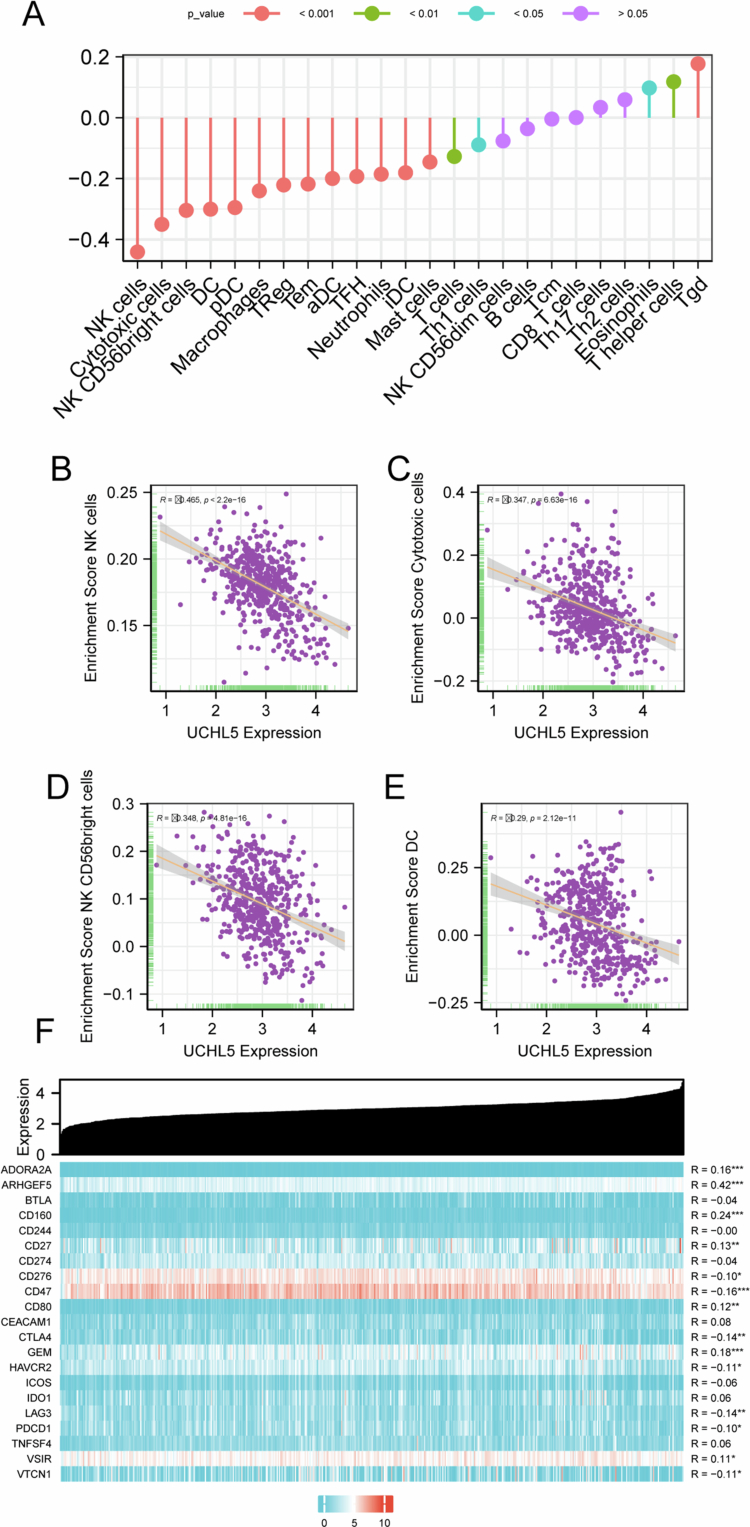
Correlation between UCHL5 expression and immune cell infiltration in THCA. (A) Bar plot showing the enrichment scores of different immune cell types (e.g., NK cells, cytotoxic T cells, macrophages, etc.) across THCA samples. (B) Scatter plot depicting the negative correlation between UCHL5 expression and NK cell enrichment scores. (C) Scatter plot showing the negative correlation between UCHL5 expression and cytotoxic T cell (CTL) enrichment scores. (D) Correlation analysis between UCHL5 expression and NK CD56 bright cell enrichment scores. (E) Correlation analysis between UCHL5 expression and dendritic cell (DC) enrichment scores. (F) Heatmap illustrating the positive correlations (R values) between UCHL5 expression and immune checkpoint markers, including PD-1 (CD274), CTLA-4, ADORA2A, and others.

Bioinformatic analysis revealed that UCHL5 expression correlated with the abundance of several immune cell populations in THCA tissues, including macrophages, neutrophils, and specific T-cell subsets (Figure 3A–E). We evaluated the relationship between UCHL5 expression and cytotoxic immune infiltration using the TIMER database. UCHL5 expression is positively correlated with ARHGEF5, CD160, and other immune checkpoint molecules (Figure 3F).

### UCHL5 expression in distinct cell types in the THCA microenvironment

**Figure 4.**
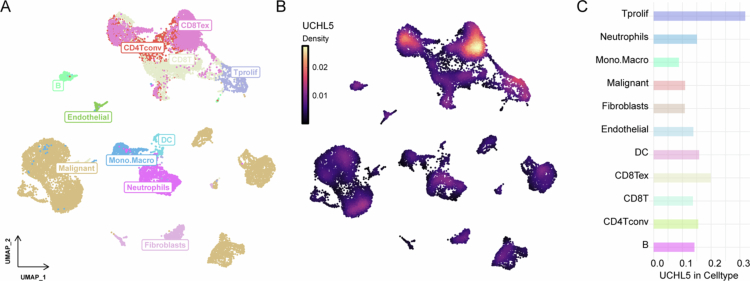
UCHL5 expression across different cell types in single-cell RNA sequencing analysis. (A) UMAP plot showing the distribution of various cell types, including CD4 T conventional (CD4Tconv), CD8 T cells (CD8T), CD8 T exhausted (CD8Tex), dendritic cells (DC), endothelial cells, fibroblasts, malignant cells, monocytes/macrophages (Mono.Macro), neutrophils, and proliferating T cells (Tprolif) in the THCA microenvironment. Each point represents an individual cell, color-coded by cell type. (B) UMAP plot illustrating the expression of UCHL5 in the same set of cell types. The expression level of UCHL5 is denoted by color intensity, with higher expression shown in darker shades. (C) Correlation analysis between UCHL5 expression and specific cell types. The plot illustrates the relative distribution of UCHL5 expression across various immune cell subsets, with a specific emphasis on CD8+ T cells, dendritic cells, and macrophages.

Single-cell RNA sequencing revealed distinct immune, stromal, and malignant cell clusters in the THCA microenvironment (Figure 4A). UCHL5 expression was cell-type specific: highest in malignant cells, fibroblasts, and monocytes/macrophages; moderate in dendritic cells and CD8+ T cells (Figure 4B,C). The expression of UCHL5 was found to be cell-type specific, being highest in malignant cells, fibroblasts, and monocytes/macrophages, and moderate in dendritic cells and CD8 T cells (Figure 4B,C).

### UCHL5 expression is associated with tumor biology and immune phenotype in THCA

**Figure 5.**
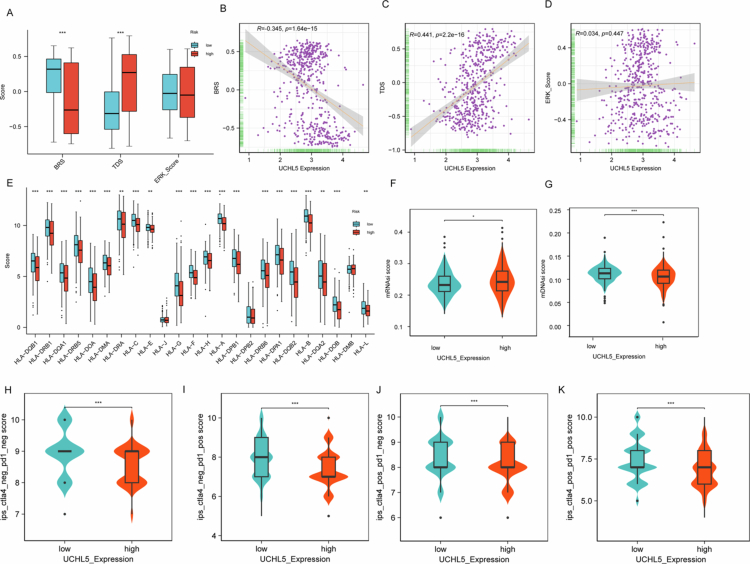
UCHL5 expression is associated with tumor biology, immunogenicity, and immune phenotypes in THCA. (A) Boxplots showing differences in basal-like score (BRS), tumor differentiation score (TDS), and ERK signaling score between UCHL5 high- and low-expression groups (*p < *0.001). (B–D) Scatter plots illustrating the correlation between UCHL5 expression and: (B) BRS (R = –0.345, *p* = 1.64e−15); (C) TDS (R = 0.441, *p* = 2.2e−16); (D) ERK score (R = 0.034, *p* = 0.447). (E) Bar plot of HLA gene expression stratified by UCHL5 level. Several MHC class II genes (e.g., HLA-DRB1, HLA-DQA1) were significantly upregulated in the high-expression group (*p < 0.05). (F, G) Tumor stemness indices in UCHL5 high- and low-expression groups: (F) mRNAsi was significantly higher; (G) mDNAsi was significantly lower in the UCHL5-high group (*p < *0.001 for both). (H–K) Immunophenoscore (IPS) comparison under different immune checkpoint blockade conditions: (H) CTLA4−/PD1−; (I) CTLA4−/PD1+; (J) CTLA4+/PD1−; (K) CTLA4+/PD1+.​​​​​

Integrated analysis revealed that UCHL5 expression was significantly negatively correlated with basal-like score (BRS) and positively correlated with tumor differentiation score (TDS), suggesting high expression of UCHL5 is associated with a more differentiated tumor phenotype with suppressed basal-like (aggressive) characteristics (Figure 5A–D). UCHL5-high tumors exhibited enhanced immunogenicity through MHC class II upregulation (Figure 5E). UCHL5-high tumors manifested a significantly increased transcriptional stemness index (mRNAsi, Figure 5F) along with a markedly diminished epigenetic stemness index (mDNAsi, Figure 5G) as compared to UCHL5-low tumors (both *p* < 0.001). Furthermore, regardless of the dimensional status of CTLA4 and PD1 blockade, CTLA4⁻/PD1⁻ (Figure 5H), CTLA4⁻/PD1⁺ (Figure 5I), CTLA4⁺/PD1⁻ (Figure 5J), and CTLA4⁺/PD1⁺ (Figure 5K), tumors with high UCHL5 showed strongly increased IPS compared to UCHL5-low tumors. This observation suggested a correlation between UCHL5 expression and THCA immune-phenotype landscape.

### UCHL5 overexpression inhibits THCA cell proliferation

**Figure 6.**
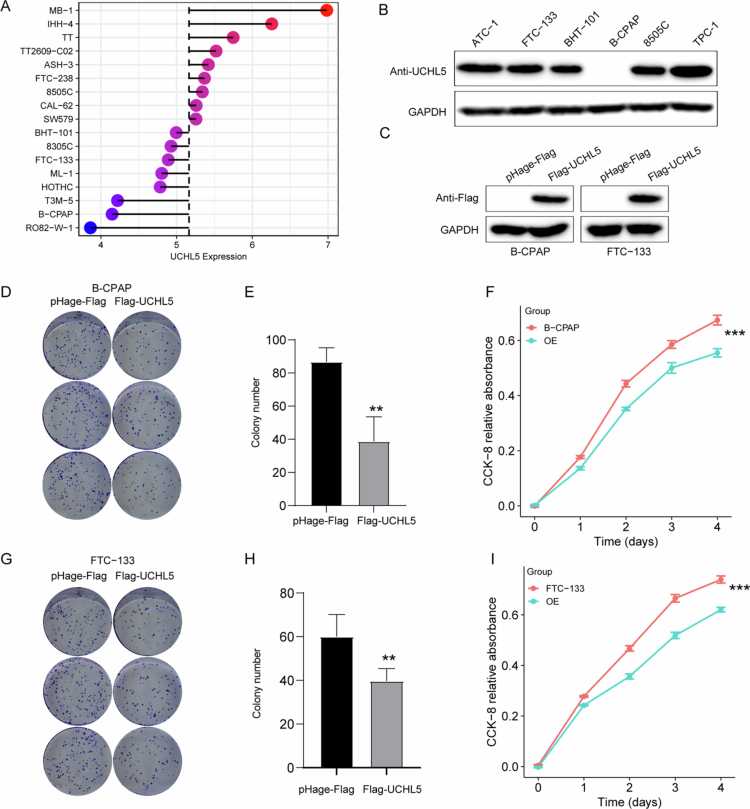
UCHL5 overexpression suppresses the proliferation of THCA cells. (A) Endogenous UCHL5 expression profile across multiple THCA cell lines. (B) Western blot analysis showing baseline levels of endogenous UCHL5 protein in 8505C, FTC-133, and B-CPAP cell lines. (C) Western blot confirming UCHL5 overexpression in FTC-133 and B-CPAP cells transfected with Flag-UCHL5 plasmids compared to control (pHage-Flag). (D, E) Clonogenic assay showing reduced colony formation in B-CPAP cells overexpressing UCHL5 (Flag-UCHL5) compared to vector control (pHage-Flag). **p < 0.01 vs pHage-Flag, unpaired two-tailed Student’s t-tests. (F) CCK-8 assay demonstrating that UCHL5 overexpression significantly inhibits the proliferation of B-CPAP cells over 4 d, ***p < 0.001 vs pHage-Flag, unpaired two-tailed Student’s t-tests. (G, H) Clonogenic assay results are consistent with (D, E), further confirming reduced colony formation in UCHL5-overexpressing B-CPAP cells, **p < 0.01 vs pHage-Flag, unpaired two-tailed Student’s t-tests. (I) CCK-8 assay validating the inhibitory effect of UCHL5 overexpression on B-CPAP cell growth at multiple time points,***p < 0.001 vs pHage-Flag, unpaired two-tailed Student’s t-tests.

To investigate UCHL5 function in THCA, we performed gain-of-function experiments. We first examined endogenous UCHL5 expression in a panel of THCA cell lines, including 8505C, FTC-133, and B-CPAP (Figure 6A). Western blot confirmed stable UCHL5 overexpression in B-CPAP and FTC-133 cells transfected with Flag-UCHL5 constructs (Figures 6C and S1). Colony formation assays on B-CPAP cells showed a significant decrease in ability to form colonies compared to vector controls (Figure 6D–E). The results of the parallel CCK-8 assay showed that B-CPAP cell lines exhibited time-dependent growth inhibition (Figure 6F). Significantly, the impaired ability of proliferation and colony formation was examined in FTC-133 cells (Figure 6G–I).

### UCHL5 knockout promotes THCA cell proliferation in vitro and in vivo

**Figure 7.**
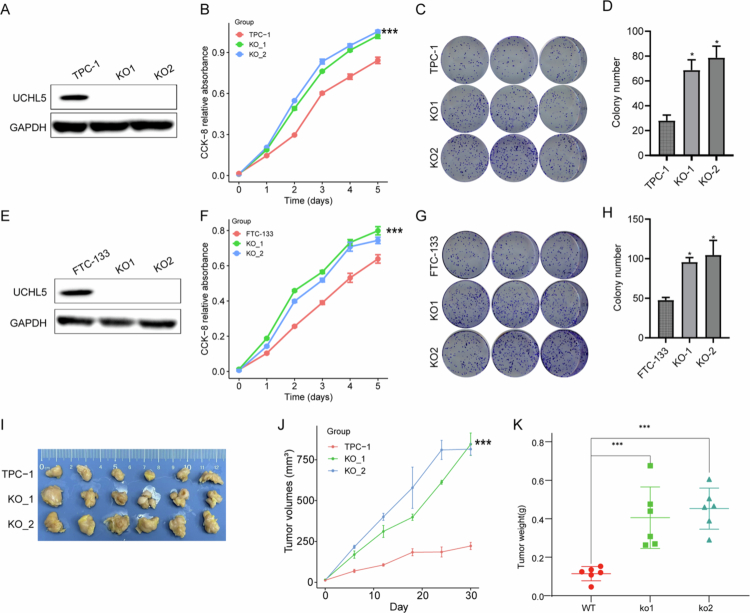
UCHL5 knockout promotes the proliferation of THCA cells in vitro and in vivo. (A) Western blot confirming effective knockout of UCHL5 protein in TPC-1 cells using CRISPR/Cas9-mediated sgRNA targeting. (B) CCK-8 assay indicating enhanced proliferation of UCHL5-KO TPC-1 cells across 5 d,***p <  0.001 vs two-way ANOVA with Tukey’s post-hoc multiple comparisons. (C, D) Colony formation assay showing increased clonogenicity in UCHL5-KO TPC-1 cells compared to control,**p < 0.01, one-way ANOVA with Tukey’s post-hoc test. (E) Western blot confirming effective knockout of UCHL5 protein in FTC-133 cells using CRISPR/Cas9-mediated sgRNA targeting. (F) CCK-8 assay indicating enhanced proliferation of UCHL5-KO FTC-133 cells across 5 d, ***p < 0.001, two-way ANOVA with Tukey’s post-hoc multiple comparisons. (G, H) Colony formation assay showing increased clonogenicity in UCHL5-KO FTC-133 cells compared to control, **p < 0.01, one-way ANOVA with Tukey’s post-hoc test. (I) Representative images of xenograft tumors from control and UCHL5-KO groups. (J) Growth curve showing significantly accelerated tumor growth in the UCHL5-KO group, ***p < 0.001, two-way ANOVA with Tukey’s post-hoc multiple comparisons. (K) Final tumor weights at endpoint demonstrate that UCHL5 knockout promotes tumor growth in vivo, **p < 0.01, one-way ANOVA with Tukey’s post-hoc test.

To further validate the tumor-suppressive role of UCHL5 in THCA, we conducted loss-of-function studies through CRISPR/Cas9-mediated knockout in multiple cell models. Western blot confirmed the depletion of UCHL5 in TPC-1 and FTC-133 cell lines (Figures 7A,E and S2). Functional assays demonstrated a considerable increase in cellular proliferation (Figure 7B,F) and clonogenic ability (Figure 7C–D, G–H) through UCHL5 knockout. Notably, in vivo xenograft assays showed that tumors deficient in UCHL5 exhibited enhanced growth, as evidenced by significantly larger tumor volumes (*p* < 0.01; Figure 7I–J) and greater end weight (Figure 7K) than wild-type controls. These in vitro and in vivo findings collectively demonstrate that UCHL5 functions as a bona fide tumor suppressor in THCA by restraining neoplastic proliferation.

### Identification of gene modules and hub genes associated with clinical traits through WGCNA in cancer

**Figure 8.**
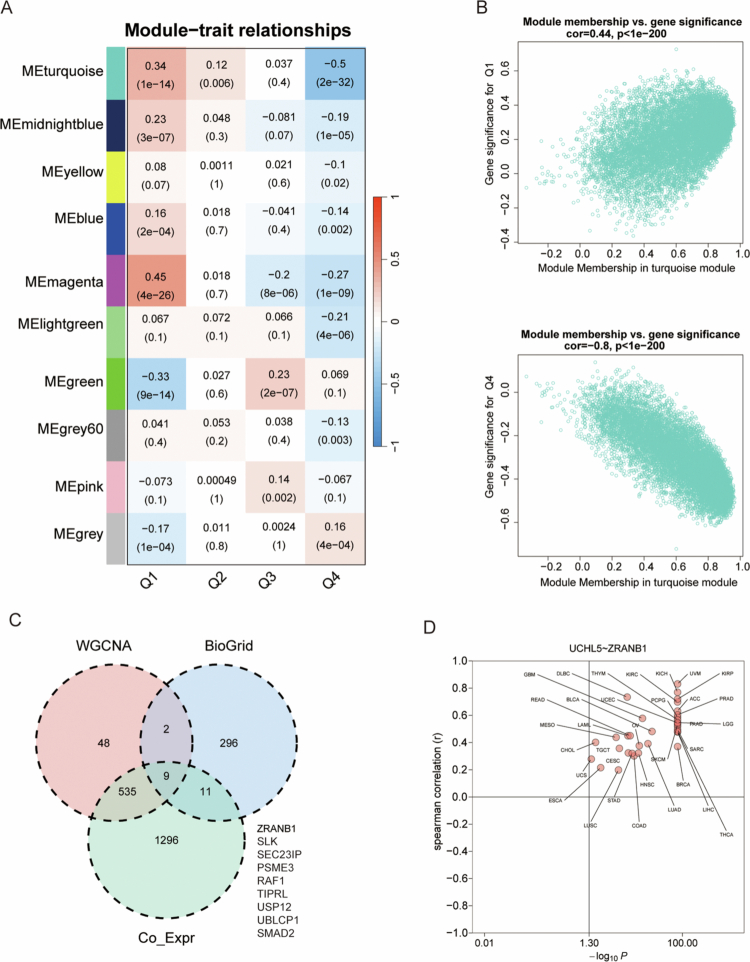
Identification of gene modules, hub genes, and expression patterns associated with clinical traits in cancer. (A) Module-trait relationships: Correlation between identified gene modules and clinical traits (Q1, Q2, Q3, and Q4). (B) Module membership vs. gene significance for Q1: The turquoise module exhibits a significant negative correlation between module membership and gene significance for trait Q1, indicating its key role in regulating gene expression linked to Q1. (C) Module membership vs. gene significance for Q4: The magenta module exhibits a significant negative correlation with trait Q4, highlighting its involvement in processes associated with Q4. (D) WGCNA BioGrid co-expression network: The co-expression network reveals hub genes, including ZRANB1, SLK, SEC23IP, PSME3, RAF1, TIPRL, USP12, and UBLCP1, which are central to the co-expression networks across multiple cancer types (e.g., BRCA, LUAD, and GBM). These genes are potential biomarkers for cancer prognosis and therapeutic targets.

WGCNA identified several gene modules associated with clinical traits in THCA (Figure 8A). Among these, the turquoise module showed the strongest negative correlation with clinical trait Q1 (Figure 8B), while the magenta module correlated negatively with Q4 (Figure 8C). A network analysis of these modules showed very significant hub genes such as ZRANB1, SLK, SEC23IP, PSME3, RAF1, TIPRL, USP12, and UBLCP1 (Figure 8D). The strong co-expression relationship between UCHL5 and ZRANB1 was of particular interest, suggesting that the two proteins may functionally interact in ubiquitin-mediated proteolysis pathways (Figure 8D).

### UCHL5 stabilizes ZRANB1 through deubiquitination

**Figure 9.**
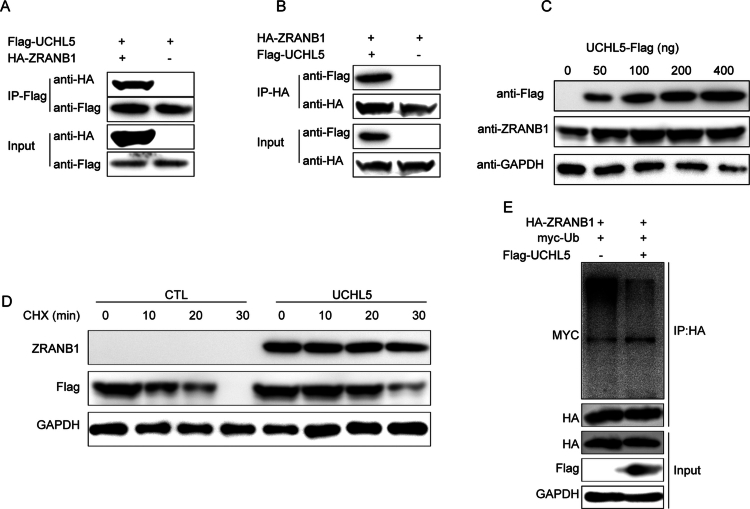
UCHL5 stabilizes ZRANB1 through direct interaction and deubiquitination in THCA. (A) Immunoprecipitation (IP) of HEK293T cells co-transfected with Flag-UCHL5 and HA-ZRANB1 for 48 h. Using anti-Flag magnetic beads to immunoprecipitate Flag-UCHL5, the co-precipitated HA-ZRANB1 was detected by Western blotting, confirming the physical interaction between Flag-UCHL5 and HA-ZRANB1. (B) Reverse co-immunoprecipitation (co-IP) experiment confirming the interaction of UCHL5 with ZRANB1 in HEK293T cells. By using anti-HA magnetic beads (or anti-HA antibody conjugated beads) to precipitate HA-ZRANB1, the co-precipitated Flag-UCHL5 was detected, which further validated the reciprocal binding between these two proteins. (C) Western blot analysis of ZRANB1 expression in HEK293T cells transfected with increasing concentrations of Flag-UCHL5 (0, 50, 100, 200, and 400 ng). (D) CHX chase assay in HEK293T cells (100 μg/ml, 0–30 min) showing the impact of UCHL5 overexpression on the half-life of ZRANB1. The results suggest that UCHL5 overexpression stabilizes ZRANB1, increasing its stability. (E) Ubiquitination assay in HEK293T cells co-transfected with HA-ZRANB1, myc-ubiquitin, and increasing amounts of Flag-UCHL5. ZRANB1 ubiquitination was detected by immunoprecipitation with anti-HA followed by immunoblotting with anti-myc.

CO-IP experiments were conducted in HEK293T cells to determine the molecular interaction of UCHL5 with ZRANB1. Reciprocal assays using anti-Flag (Figure 9A) and anti-HA (Figure 9B) antibodies revealed direct physical interaction between these proteins. The interaction was found to have functional significance as UCHL5 overexpression resulted in a dose-dependent increase in ZRANB1 protein levels (Figure 9C). Protein stability assays indicated that UCHL5 increased the half-life of ZRANB1 in cycloheximide (CHX) chase experiments (Figure 9D), suggesting post-translational regulation. Ubiquitination assays demonstrated that UCHL5 stabilizes ZRANB1 by decreasing its ubiquitination, as evidenced by reduced myc-ubiquitin signal (Figure 9E) when UCHL5 was co-expressed. The finding was further supported by dose-response experiments showing that ZRANB1 ubiquitination was lower in a UCHL5-dependent manner.

### UCHL5 drives ferroptosis sensitivity by stabilizing ZRANB1

**Figure 10.**
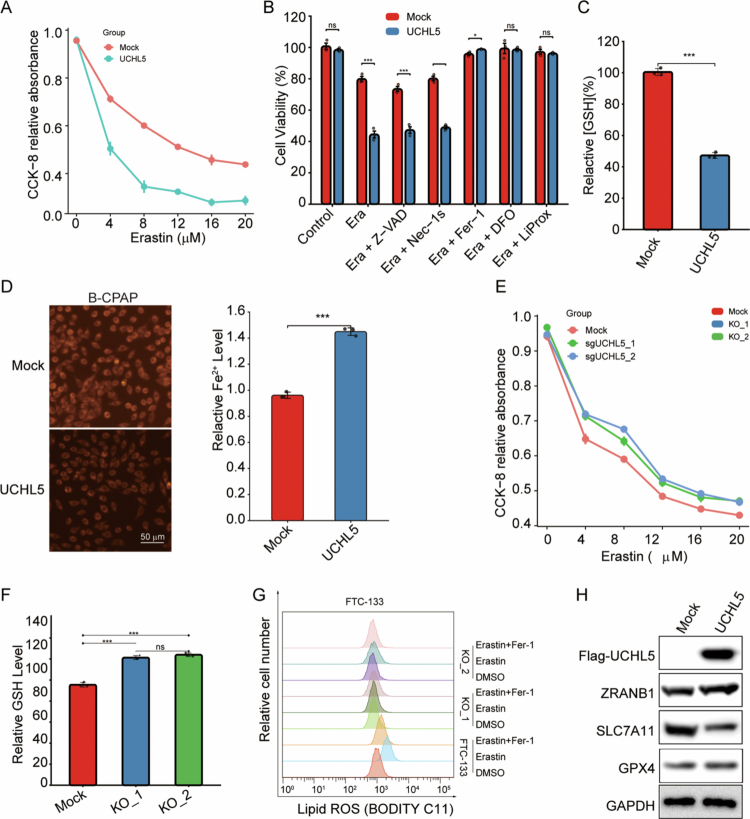
UCHL5 regulates ferroptosis sensitivity in THCA cells via the ZRANB1–SLC7A11 axis. (A) Cell viability in B-CPAP stable cell lines (UCHL5 overexpression) after treatment with different concentrations of Erastin, ***p < 0.001, one-way ANOVA with Tukey’s post-hoc multiple comparisons. (B) Bar graphs showing cell viability in B-CPAP stable cell lines (UCHL5 overexpression) treated with 20 μM Erastin combined with 5 μM Z-VAD-fmk (Z-VAD), 2 μM Necrostatin-1s (Nec-1s), 2 μM Fer-1, 100 μM DFO, or 10 μM Liprox, ***p < 0.001 vs corresponding control, one-way ANOVA with Tukey’s post-hoc test. (C) Bar graph showing intracellular GSH levels in the indicated B-CPAP stable cell lines (UCHL5 overexpression), ***p < 0.001 vs Mock, unpaired two-tailed Student’s t-tests. (D) Intracellular ferrous ion (Fe²⁺) levels in UCHL5-overexpressing B-CPAP cells were detected by FerroOrange fluorescent probe. Images were captured using an automated cell imaging analysis system, and fluorescence intensity was quantified via ImageJ (*n* = 3). Scale bar: 50 μm, ***p < 0.001 vs Mock, unpaired two-tailed Student’s t-tests. (E) CCK-8 assay showing decreased viability of FTC-133 cells following UCHL5 knockout (using sgUCHL5_1 and sgUCHL5_2) upon Erastin treatment, ***p < 0.05, one-way ANOVA with Tukey’s post-hoc multiple comparisons. (F) Bar graph showing intracellular GSH levels in FTC-133 cells with UCHL5 knockout (sgUCHL5_1 and sgUCHL5_2), ***p < 0.001, one-way ANOVA with Tukey’s post-hoc test. (G) Flow cytometry analysis of lipid ROS (using BODIPY C11) in FTC-133 cells with UCHL5 knockout. (H) Western blot analysis of ZRANB1, SLC7A11, and GPX4 expression in UCHL5-overexpressing cells compared to Mock controls.

To investigate the functional role of UCHL5 in ferroptosis regulation, we performed both gain- and loss-of-function studies in FTC-133 THCA cells. UCHL5 overexpression significantly enhanced cellular sensitivity to the ferroptosis inducer erastin (0–20 μM), as demonstrated by a marked reduction in cell viability compared to control cells (*p* < 0.001; Figure 10A). Notably, inhibitor co-treatment assays (Figure 10B) showed that among different treatment groups (Control, Era+Z-VAD, Era, Era+Nec-1s, Era+Fer-1, Era+DFO, Era+LiProx), the decreased cell viability in UCHL5-overexpressing cells upon erastin treatment could only be rescued by ferroptosis-related inhibitors (ferrostatin-1 [Fer-1], deferoxamine [DFO], and Liproxstatin-1 [LiProx]), while the apoptosis inhibitor Z-VAD and necroptosis inhibitor Nec-1s had no significant effect (Figure 10B), confirming that UCHL5 regulates cell death specifically through ferroptosis. Consistent with this, the intracellular glutathione (GSH) content in UCHL5-overexpressing cells was significantly lower than that in the Mock group (Figure 10C), indicating that UCHL5 impairs the cellular antioxidant capacity, which is critical for resisting ferroptosis. We further validated the regulatory effect of UCHL5 on iron homeostasis in B-CPAP cells: using FerroOrange fluorescent probe combined with automated cell imaging and ImageJ quantitative analysis, we found that UCHL5 overexpression could significantly increase the intracellular ferrous ion (Fe²⁺) level in B-CPAP cells (Figure 10D), which provides the iron overload required for ferroptosis.

On the contrary, CRISPR/Cas9-induced knockout of UCHL5 with two independent sgRNAs, sgUCHL5_1 and sgUCHL5_2, robustly conferred resistance to ferroptosis, as UCHL5-deficient cells had significantly higher viability to erastin treatment (*p* < 0.01; Figure 10E). The impact of UCHL5 depletion on cellular antioxidant capacity was further evaluated (Figure 10F). Both UCHL5 knockout clones (KO_1 and KO_2) exhibited significantly elevated GSH levels compared to Mock controls (Figure 10F). Furthermore, BODIPY C11 flow cytometric determination results showed that UCHL5 knockout greatly diminished lipid ROS accumulation induced by erastin, which could be rescued by the ferroptosis inhibitor ferrostatin-1 (Fer-1, 1 μM; Figure 10G). Moreover, the UCHL5 overexpression enhanced the expression level of ZRANB1 but suppressed the level of SLC7A11 and GPX4, which are the key suppressors of ferroptosis, as shown by Western blot analysis (Figure 10H).

## Discussion

Recent studies suggest that UCHL5 is involved in cancer biology in a context-dependent manner. Specifically, UCHL5 functions as either an oncogene or a tumor suppressor. UCHL5 works to deubiquitinate and stabilize *β*-catenin, which enables the activation of glycolysis through the Wnt/β-catenin pathway in hepatocellular carcinoma.^8^ UCHL5 stabilizes ELK3 and enhances Notch1-mediated stemness, which drives pancreatic ductal adenocarcinoma progression.^18^ Nevertheless, our data show that reduced expression of UCHL5 is associated with the advanced tumor stage and lymph node metastasis, indicating its potential as a biomarker for progression. Functionally, UCHL5 overexpression suppressed tumor cell proliferation, whereas knockout models exhibited enhanced growth in vitro and in vivo, suggesting that UCHL5 plays an anti-tumor role in THCA. The context-specific function of UCHL5 might be related to the dysregulation of UCHL5 in different cancers.

The functional versatility of UCHL5 is largely determined by its deubiquitinase activity to stabilize different substrates, such as ELK3,^9^ NFRKB,^19^ and *β*-catenin.^12^ Here, we discovered ZRANB1 as a novel and functionally relevant substrate of UCHL5 in the context of THCA. ZRANB1, a deubiquitinase targeting K63-linked polyubiquitin chains, was recently reported to function as an E3 ligase for SLC7A11, thereby regulating ferroptosis resistance.^20^ While ZRANB1 has been documented to promote progression in cancers, hepatocellular carcinoma,^21^ and colorectal cancer.^22,^^23,^^24^ Conversely, our data showed that UCHL5 overexpression increased ZRANB1 expression, resulting in the downregulation of SLC7A11/GPX4. These data suggest that UCHL5 interacts with ZRANB1 and activates ferroptosis. Therefore, our work not only identifies a new substrate for UCHL5 but also delineates a previously unreported UCHL5-ZRANB1-SLC7A11/GPX4 regulatory axis that governs ferroptosis in THCA.

In summary, here we identify UCHL5 as a critical tumor suppressor in thyroid cancer, functioning through dual mechanisms: it stabilizes ZRANB1 via deubiquitination and enhances ferroptosis sensitivity by downregulating the SLC7A11-GPX4 axis. These findings establish a previously unrecognized tumor-suppressive pathway in THCA. Despite these contributions, this study has several limitations. Initially, we note that our immune-related analyses are bioinformatic and correlate, not functional analyses. Future research on the function of UCHL5 in remodeling the TME should use immune cell co-culture systems and immunocompetent animal models. Moreover, we did not investigate the function of UCHL5 in different THCA subtypes (e.g., papillary versus follicular THCA) or its interaction with the BRAF major driver mutation, which would help to further define the clinical relevance. Finally, survival analysis was not possible due to incomplete follow-up data from our clinical cohort. Thus, the prognostic value of UCHL5 needs to be confirmed in a large long-term cohort study. Despite these limitations, our findings establish the UCHL5-ZRANB1 axis as a potential therapeutic target in THCA.

## Supplementary Material

Supplementary material Supplementary figure captions.

Fig S1.tif Fig S1.tif

Fig S2.tif Fig S2.tif

## Data Availability

Publicly available datasets (TCGA, GEO) were analyzed in this study and can be accessed via the methods described. All data generated or analyzed during this study are included in this manuscript and its supplementary information files.
