# Supplementary material for: UCHL5 suppresses thyroid carcinoma progression via ZRANB1 stabilization and ferroptosis regulation
Source: Cancer Biol Ther. 2026 Apr 27;27(1):2663610. doi: 10.1080/15384047.2026.2663610 (PMC13123060; doi:10.1080/15384047.2026.2663610)
Supplement: Supplementary material — Supplementary figure captions. [file KCBT_A_2663610_SM5377.docx]

Fig S1. Western blot analysis of UCHL5 overexpression in B-CPAP and FTC-133.

Fig S2 Western blot analysis of UCHL5 knockout in B-CPAP and FTC-133.
