# Supplementary figures and images for: UCHL5 suppresses thyroid carcinoma progression via ZRANB1 stabilization and ferroptosis regulation
Source: Cancer Biol Ther. 2026 Apr 27;27(1):2663610. doi: 10.1080/15384047.2026.2663610 (PMC13123060; doi:10.1080/15384047.2026.2663610)

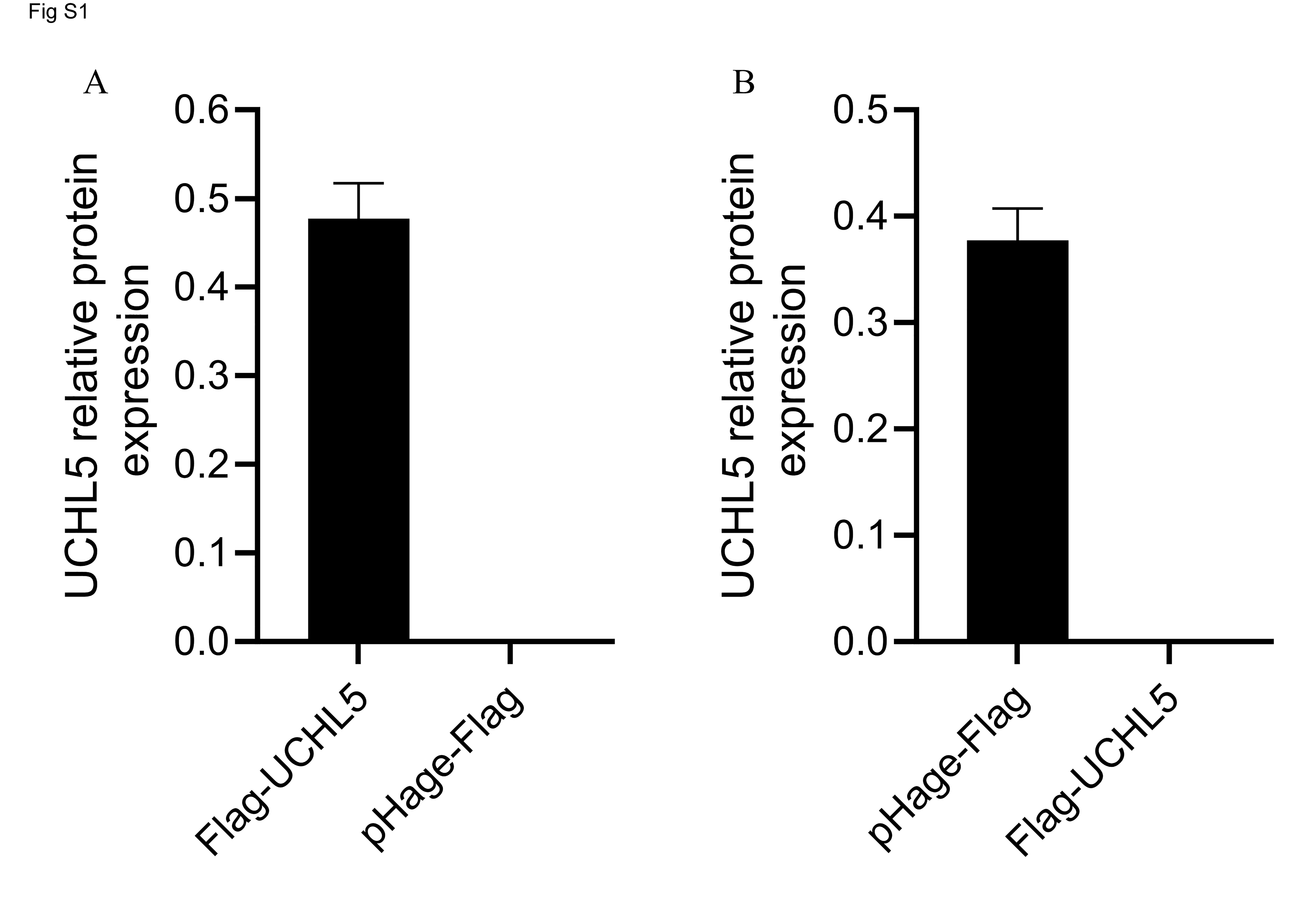

Supplement: Fig S1.tif [file KCBT_A_2663610_SM4197.tif]

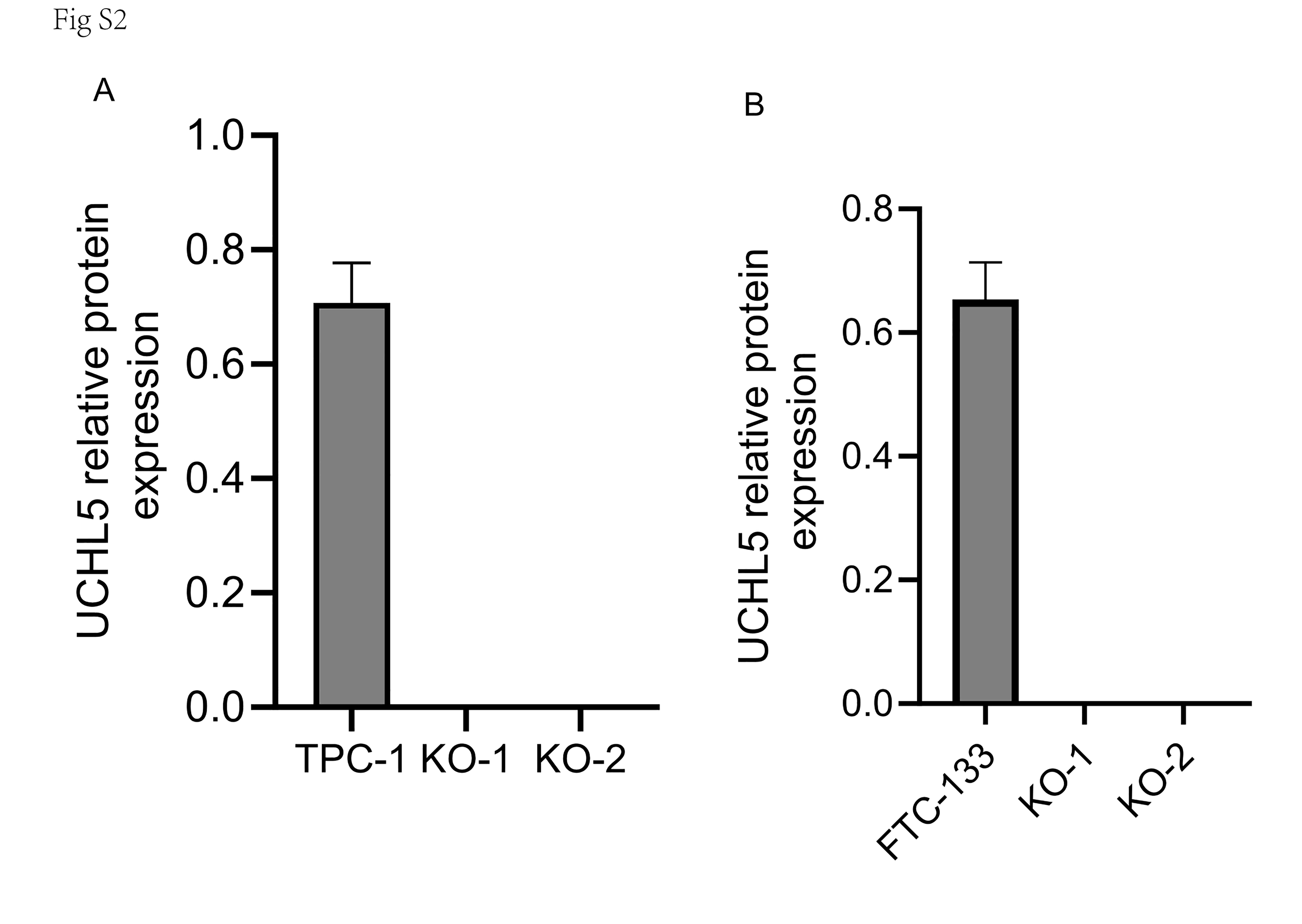

Supplement: Fig S2.tif [file KCBT_A_2663610_SM4196.tif]
